# Assessment of an Enterobactin Conjugate Vaccine in Layers to Protect Their Offspring from Colibacillosis

**DOI:** 10.3390/pathogens12081002

**Published:** 2023-07-31

**Authors:** Huiwen Wang, Catherine M. Logue, Lisa K. Nolan, Jun Lin

**Affiliations:** 1 Department of Animal Science, University of Tennessee, Knoxville, TN 37919, USA; 2 Department of Population Health, College of Veterinary Medicine, University of Georgia, Athens, GA 30602, USA; 3 Department of Infectious Diseases, College of Veterinary Medicine, University of Georgia, Athens, GA 30602, USA

**Keywords:** enterobactin, maternal immunization, immunoglobulin Y, avian pathogenic *E. coli*

## Abstract

Colibacillosis, caused by avian pathogenic *Escherichia coli* (APEC), is an important infectious disease in chickens and a major cause of mortality in young chicks. Therefore, protecting young chickens from colibacillosis is important for improving welfare and productivity in the poultry industry. Recently, we developed a novel enterobactin (Ent) conjugate vaccine that could induce high titers of anti-Ent immunoglobulin Y (IgY) in chicken serum and consequently mitigate the organ lesions caused by APEC infection. Considering that maternal immunization is a practical approach to confer instant immune protection to the hatchlings, in this study, we immunized breeder hens with the Ent conjugate vaccine and evaluated the maternal immune protection on the progenies challenged with APEC. Three doses of the vaccine induced high titers of anti-Ent IgY in the hens (about 16- and 64-fold higher than the control group in the sera and egg yolks, respectively), resulting in an eight-fold of increase in anti-Ent IgY in the sera of progenies. However, the anti-Ent maternal immunity did not display significant protection against APEC challenge in the young chicks as there was no significant difference in APEC load (in liver, lung, and spleen) or organ lesions (in heart, liver, spleen, lung, and air sac) between the vaccinated and control groups. In future studies, the APEC infection model needs to be optimized to exhibit proper pathogenicity of APEC, and the maternal immunization regimen can be further improved to boost the maternally derived anti-Ent IgY in the hatchlings.

## 1. Introduction

Colibacillosis is a complex of localized and systemic infections that are caused by avian pathogenic *Escherichia coli* (APEC) and result in early mortality, condemnation of carcasses, and reduced productivity in poultry [1]. Particularly in young chickens, APEC constitutes a major cause of early mortality [2]. Thus, protecting young chickens from colibacillosis is important for improving welfare and productivity in the poultry industry. Maternal antibodies (immunoglobulin Y, IgY) in young birds are known to be protective against different infectious agents, including bacteria, viruses, and parasites [3,4,5]. Maternal antibodies are vertically transferred from breeder hens to their progeny during egg formation and can persist in the chick’s circulation for up to 3 weeks after hatching [6]. Therefore, immunization of breeder hens may confer passive immune protection against APEC infection in young chicks. This maternal immunization strategy has been widely tested for controlling infectious diseases in poultry, such as necrotic enteritis [7,8], salmonellosis [9,10], and coccidiosis [11,12], and has shown promising outcomes. In particular, immunization of broiler breeders has become a common practice to control salmonellosis in the offspring.

Enterobactin (Ent) is an archetypical siderophore (iron chelator) produced by members of the Enterobacteriaceae family including *E. coli*, and can efficiently scavenge nutrient iron for bacterial pathogens from the hosts [13]. Prior studies have shown that Ent-mediated iron acquisition plays an important role in the fitness and pathogenesis of different pathovars of *E. coli*, such as APEC and uropathogenic *E. coli* [14,15]. To counteract the iron sequestration by Ent during bacterial infection, the mammalian host secretes an acute phase protein named lipocalin-2 to tightly bind Ent and consequently suppress the bacterial infection [16,17]. The bacteriostatic effect of lipocalin-2 suggests that directly targeting Ent to develop immune interventions can be a promising antimicrobial strategy to control *E. coli* and other Ent-dependent bacterial pathogens. This innovative concept was materialized in our recent studies, in which we developed novel Ent conjugate vaccines that could induce high titers of Ent-specific antibodies in rabbits and chickens [18,19], and the anti-Ent antibodies could significantly inhibit in vitro growth of different pathovars of *E. coli*, including various APEC strains [20]. Further, active immunization of chickens with the Ent conjugate vaccine was demonstrated to significantly mitigate the organ lesions caused by APEC infection [21].

However, this active immunization regimen needs a few weeks to mount a sufficient level of protective antibodies and cannot provide instant immune protection for young chicks that are especially vulnerable to APEC infection. Considering that maternal immunity can protect young chicks from microbial infections, in this study, we immunized breeder hens with the Ent conjugate vaccine and evaluated the protective effect on the progeny challenged with APEC.

## 2. Materials and Methods

### 2.1. Preparation of Ent Conjugate Vaccines

An Ent transport mutant of *E. coli* AN102 (courtesy of Dr. Sandra Armstrong at the University of Minnesota) was used for Ent purification as described previously [22,23]. The purified Ent was conjugated to bovine serum albumin (BSA, Cat. No. PI77110, Fisher Scientific, Rockford, IL, USA) or keyhole limpet hemocyanin (KLH, Cat. No. PI77600, Fisher Scientific, Rockford, IL, USA) using the method described in our previous publication [18]. To validate the conjugation, the KLH–Ent and BSA–Ent conjugates were subjected to immunoblotting analysis using a highly specific anti-Ent monoclonal antibody generated in our recent study [24]. Lastly, the Ent conjugates (1 mg/mL in PBS) were stored at −20 °C prior to use.

### 2.2. Immunization of Breeder Hens

The chicken experiment was approved by Institutional Animal Care and Use Committee at the University of Georgia (IACUC No. 2018-01-014 and 2021-02-008). Twelve breeder hens (Barred Rock) at 20 weeks of age were evenly assigned into 2 groups with one rooster (Rhode Island Red) being introduced to each group for fertilization. The chickens were housed in a clean laboratory setting with a constant temperature of 20 °C and a photoperiod of 14 h. The hens in the treatment group were subcutaneously (neck) immunized with 100 µL of KLH–Ent (100 µg per bird) emulsified in Freund’s complete adjuvant (Thermo Fisher, Rockford, IL, USA), followed by two booster immunization (100 µg of KLH–Ent emulsified in Freund’s incomplete adjuvant (Thermo Fisher, Rockford, IL, USA)) with a 2-week interval. Meanwhile, the other group served as non-immunization control. To examine the vaccine-specific IgY level in the hens, serum and egg of each hen were collected upon the first immunization and two weeks after the third immunization. The samples were subjected to enzyme-linked immunosorbent assay (ELISA) as described below. In addition, the fertilized eggs collected within the second week after the last immunization were subjected to synchronous hatching with a standard incubator setting [25]. Briefly, the incubation was maintained at a temperature of 37.5 °C and a relative humidity of 60%. The turning time interval during incubation was 2 h until day 18. The eggs were examined by candling on day 18, and those with a living embryo were transferred to hatching baskets.

### 2.3. APEC Challenge of Hatchlings

The two groups of newly hatched chicks (28 and 31 birds in control and vaccination groups, respectively) were housed separately in a specific-pathogen-free setting with a constant temperature of 34 °C and a photoperiod of 12 h. At 5 days of age, sera were collected from 6 representative chicks in each group to examine the vaccine-specific IgY level. In the meantime, all the chicks in both groups were intratracheally challenged with APEC O78 (10^7^ CFU per bird), an O78:K80:H9 strain (χ7122, courtesy of Dr. Roy Curtiss III at the University of Florida) originally isolated from the liver of a diseased turkey [26]. Two days post challenge, all the chicks were humanely euthanized using a method approved by the American Veterinary Medical Association. Necropsy were conducted to examine the lesions in lung, air sac, heart, liver, and spleen. Lesion scores were graded independently by two veterinarians using a scoring system described by Antão et al. [27]. To assess the APEC load in lung, liver, and spleen, the tissues were aseptically removed and placed in sterile Whirl-Pak® bags on ice. At the laboratory, the organs were weighed and homogenized in sterile PBS. Serial dilutions of the homogenized tissue were then plated on MacConkey agar. After overnight incubation at 37 °C, the colonies on plates were enumerated and converted to bacterial load in tissue (CFU/g).

### 2.4. ELISA

The specific antibody level in chicken serum and egg yolk was measured by indirect ELISA as described in our previous publications [19,21]. Briefly, microtiter plates (Nunc-MaxiSorp™ Plate, Thermo Fisher, Rochester, NY, USA) were coated with appropriate antigens (detailed below) in 100 μL of coating buffer (sodium bicarbonate/carbonate buffer, pH 9.6) overnight at room temperature. Specifically, to determine the antibody levels against the whole KLH–Ent conjugate, KLH–Ent conjugate (30 ng/well) was used to coat ELISA plates. To determine the antibody level against the molecule Ent, BSA–Ent (2 µg/well) was used to coat ELISA plates. The coated plates were blocked with blocking buffer (PBS containing 0.05% Tween 20 and 5% skim milk) for 1 h at room temperature. Subsequently, chicken serum and egg yolk samples were 2-fold serially diluted in blocking solution and incubated with the coated plates for 1 h at room temperature. Following four washes with washing buffer (PBS containing 0.05% Tween 20), horseradish peroxidase-conjugated goat anti-chicken-IgY secondary antibody (SeraCare, Milford, MA, USA) with 2000-fold dilution in blocking buffer was added, and the plates were incubated at room temperature for 1 h. After another four washes with washing buffer, the plates were finally developed using an ABTS peroxidase substrate (SeraCare, Milford, MA, USA). The reaction was stopped after 30 min by adding 100 µL of stop solution (1% SDS). The absorbance was measured at the optical density of 405 nm (OD_405_) using a microplate reader (BioTek Instruments, Winooski, VT, USA), and the data were collected using Gen5 software (BioTek Instruments). The wells without addition of the primary antibody served as background control. Endpoint titer was defined as the last dilution at which OD_405_ of the sample wells exceeded the cutoff value (0.1). Duplicate measurements were performed for each sample.

### 2.5. Statistical Analysis

The pairwise comparisons between the control and vaccination groups were conducted using statistical methods as appropriate. A probability level of *p* < 0.05 was considered statistically significant.

## 3. Results

### 3.1. Validation of the Ent Conjugates

**Figure 1:**
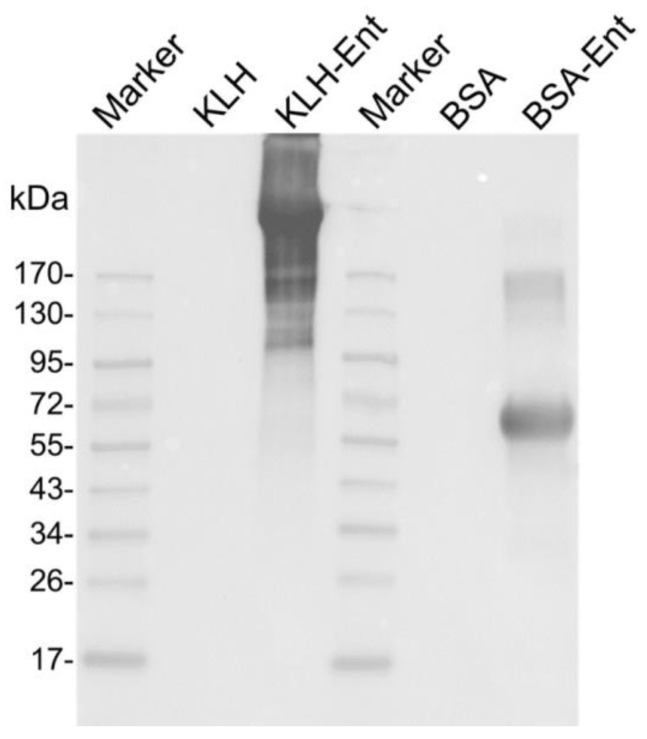
Validation of the Ent conjugates. Western blotting of KLH, KLH–Ent, BSA, and BSA–Ent detected by the anti-Ent monoclonal antibody.

As shown in Figure 1, the anti-Ent monoclonal antibody generated in our recent study [24] could vividly react with KLH–Ent or BSA–Ent conjugate, resulting in a strong smear for KLH–Ent or a single band for BSA–Ent. Meanwhile, the anti-Ent monoclonal antibody did not react with the carrier protein KLH or BSA alone. The KLH–Ent and BSA–Ent conjugates were successfully produced and could be used for subsequent immunization and immunoassays.

### 3.2. Immune Responses in the Hens and Hatchlings

**Figure 2:**
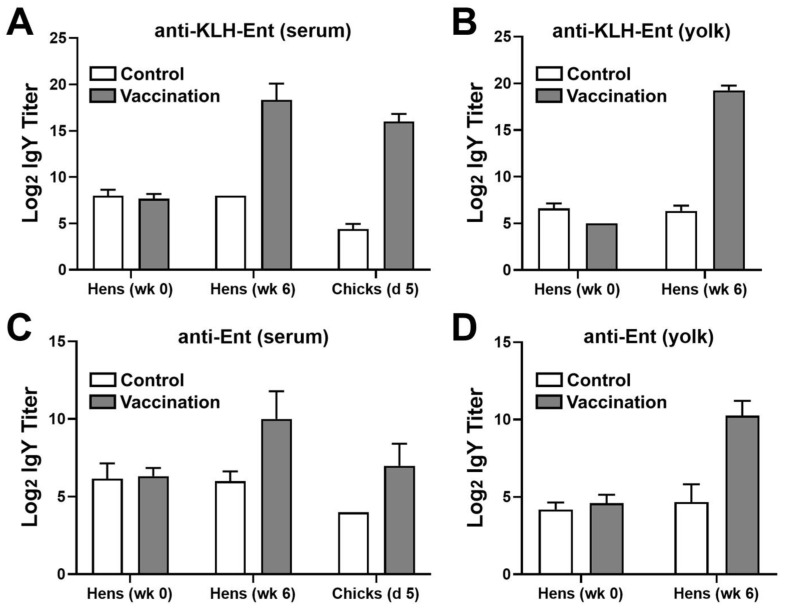
KLH–Ent vaccine induced high titers of specific IgY in the hens’ sera and egg yolks as well as in the progenies’ sera. (**A**) Specific IgY against the whole KLH–Ent conjugate in the sera of hens and chicks. (**B**) Specific IgY against the whole KLH–Ent conjugate in the egg yolks of hens. (**C**) Ent-specific IgY in the sera of hens and chicks. (**D**) Ent-specific IgY in the egg yolks of hens. For hens, weeks 0 and 6 represent the time points of first vaccination and 2 weeks after the third vaccination. For chicks, day 5 represents their age when they were intratracheally challenged with APEC. The data are presented as mean ± SD.

Immunization of hens with the KLH–Ent conjugate induced high levels of antibody response in the sera and egg yolks, and maternal immunity was successfully transferred to the hatched chicks (Figure 2). Specifically, compared to the unvaccinated hens, the vaccinated hens displayed over 10 log_2_ units (1024-fold) higher titers of anti-KLH–Ent IgY in the sera 2 weeks after the third dose of KLH–Ent vaccine (Figure 2A). In the egg yolks, the vaccination induced 13 log_2_ units (8192-fold) of increase in anti-KLH–Ent IgY when compared to the unvaccinated group (Figure 2B). As a result, the specific maternal immunity was transferred to the progeny, reflected by about 12 log_2_ units (4096-fold) of increase in anti-KLH–Ent IgY in the sera of immunization group relative to the control group at 5 days of age (Figure 2A).

Similarly, high titers of Ent-specific IgY were also induced in the vaccinated hens, which exhibited approximately 4 and 6 log_2_ units (16- and 64-fold) of increase in the sera and egg yolks, respectively, when compared to the unvaccinated hens 2 weeks after the third dose of immunization (Figure 2C,D). Consequently, the progeny in immunization group, compared to the control group, had 3 log_2_ units (8-fold) higher titers of anti-Ent IgY in the sera 5 days after hatching (Figure 2C).

### 3.3. Organ Lesions Caused by APEC Challenge

**Figure 3:**
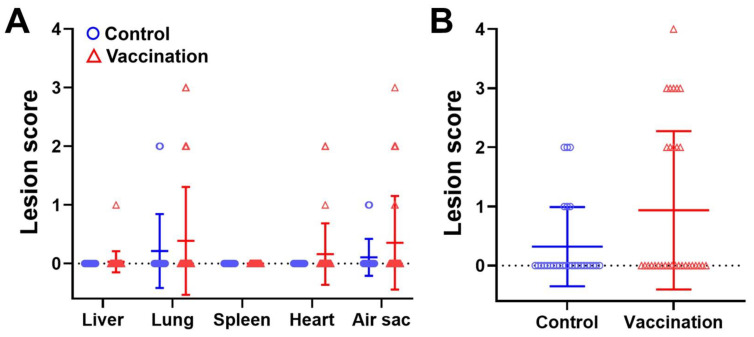
Organ lesions in the chicks challenged with APEC. (**A**) Lesion scores in the five different organs of chicks at 2 days post-infection (7 days of age). (**B**) Accumulative lesion scores of the five organs at 2 days post-infection (7 days of age). The data are presented as mean ± SD. Mann–Whitney *U* test was used for statistical analysis.

To evaluate the protective effect of the Ent-specific maternal immunity, the chicks were humanely euthanized at 7 days of age (2 days post-infection) and examined for organ lesions caused by the APEC challenge. Only a small portion of the chicks in both immunization and control groups exhibited visible lesions in the liver, lung, spleen, heart, or air sac, and the lesion scores in each organ were statistically comparable between the two groups (Figure 3A). When the lesion scores of the five organs were accumulated, there was still no significant difference (Figure 3B).

### 3.4. APEC Load in the Organs

**Figure 4:**
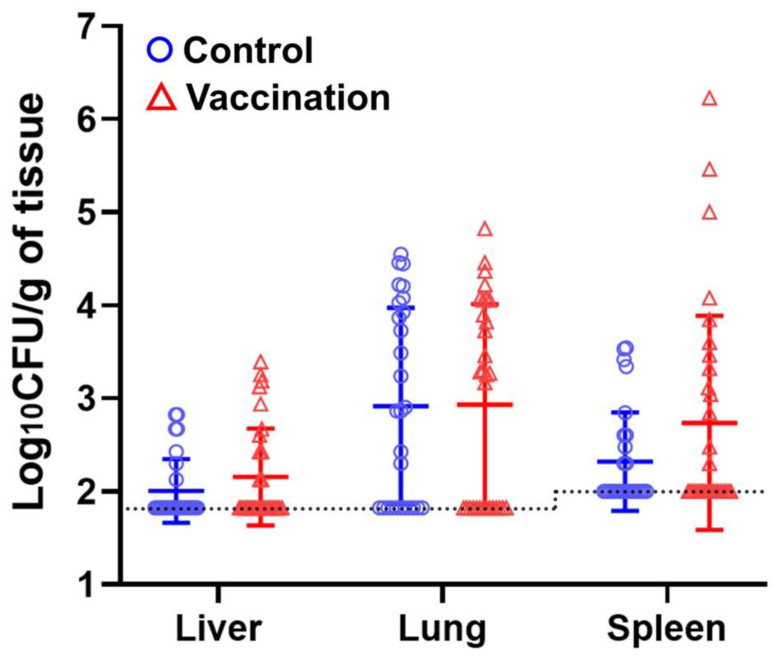
APEC load in the organs of infected chicks. The dashed line indicates detection limits (67 CFU/g for liver and lung; 100 CFU/g for spleen). The data are presented as mean ± SD. Mann–Whitney *U* test was used for statistical analysis.

APEC load in the liver, lung, and spleen of chicks was also examined to assess immune protection conferred by the anti-Ent maternal immunity. Similar to the organ lesions, there was no statistical difference in APEC burden in the 3 organs between immunization and control groups (Figure 4). Notably, many of the chicks had a low level of APEC infection that was below the detection limit (67 CFU per gram of liver and lung tissues, 100 CFU per gram of spleen tissue) (Figure 4). This was consistent with the finding that majority of the chicks had no observable lesions in their organs (Figure 3B).

## 4. Discussion

Ent-mediated iron acquisition is conserved in many Gram-negative bacterial pathogens, such as *E. coli*, *Salmonella enterica*, and *Klebsiella pneumoniae*, and plays an important role in their fitness and pathogenesis [14,28,29,30]. In addition, other bacterial pathogens like *Campylobacter jejuni* and *Pseudomonas aeruginosa*, although not producing Ent, can take up exogenous Ent to gain a fitness advantage (siderophore piracy) [31,32]. Thus, the Ent system has been an attractive target for developing antimicrobial strategies over the past few decades, such as inhibitors for Ent biosynthesis and vaccines against outer-membrane Ent receptors [33,34]. However, these approaches have had limited success, primarily due to the complexity and redundancy of systems for synthesizing and transporting Ent. For example, up to five Ent receptors (i.e., FepA, Cir, Fiu, IroN, and Iha) have been identified in different *E. coli* strains [33]. The bacteriostatic effect of host lipocalin-2 directly binding Ent inspired us to directly target the Ent molecule for developing innovative immune interventions. In our recent studies, we successfully generated Ent conjugate vaccines and optimized the vaccination regimens to induce high titers of anti-Ent antibodies in chickens [18,19]. Further, active immunization of chickens with the Ent conjugate vaccine was demonstrated to significantly repress the intestinal colonization of *C. jejuni* or alleviate the organ lesions cause by APEC [21,35]. These studies have laid a solid foundation for us to assess protective efficacy of the maternally derived anti-Ent immunity, a practical approach to protect young chicks from APEC infection.

However, despite high titers of anti-Ent antibodies induced in the immunized hens’ sera and egg yolks as well as the progenies’ sera (Figure 2), the anti-Ent maternal immunity failed to confer protection against APEC challenge in young chicks as it did not significantly reduce APEC load or lesions in the chicks’ organs (Figure 3 and Figure 4). We reason some possibilities that may explain why no immune protection was observed. In our recent study, active immunization of chickens with the Ent conjugate vaccine induced a 6 log_2_ units (64-fold) of increase in the anti-Ent IgY titer, which exerted a significant amelioration on the organ lesions caused by APEC infection [21]. Meanwhile in this study, the maternally derived anti-Ent IgY in the young chicks was 3 log_2_ units (8-fold) higher compared to the control group. Thus, we speculate that despite a significant level of anti-Ent maternal IgY in the chicks’ sera, the specific antibody titer was not sufficiently high to protect the chicks from APEC infection.

A notable limitation of this study is that more than half of the chicks in control group had undetectably low levels of APEC in their organs and did not exhibit visible organ lesions. This suggests that the APEC infection model is not yet optimal for evaluating the maternal immune efficacy. Since the breeder hens used in this study were obtained from a commercial breeding farm, it is possible that the hens had preexisting immunity against APEC due to routine immunization with APEC vaccine or exposure to APEC infection before they were enrolled in this study. In this case, the APEC-specific immunity might be transferred from the hens to their progeny and, to some extent, suppress APEC infection in the chicks. Alternatively, this particular breed of chicks (a hybrid of Barred Rock female and Rhode Island Red male) might be genetically less susceptible to APEC infection despite a high challenge dose (10^7^ CFU per bird) of APEC O78 strain exhibiting strong pathogenicity in previous studies [26,36]. Thus, in future studies, we will optimize the APEC infection model by excluding preexisting immunity against APEC in breeder hens and identifying breeds of chicks with proper susceptibility to APEC infection. Importantly, we will also optimize the Ent-based immunization regimen to further improve maternally derived anti-Ent IgY titer in the hatchlings. By taking these optimization measures, we are hopeful to see the Ent-based maternal immunization protecting the young chicks from colibacillosis.

## 5. Conclusions

In this study, immunization of breeder hens with the Ent conjugate vaccine induced high titers of anti-Ent IgY in the hens’ sera and egg yolks as well as in the sera of progenies. Nevertheless, the anti-Ent maternal immunity did not display significant protection against APEC challenge in the young chicks as it did not mitigate the APEC load and organ lesions. In future studies, the APEC infection model needs to be optimized to exhibit proper pathogenicity of APEC, and the maternal immunization regimen can be further improved to boost the maternally derived anti-Ent IgY in the hatchlings.

## Data Availability

The data presented in this study are available on request from the corresponding author.

## References

[1] Kathayat D., Lokesh D., Ranjit S., Rajashekara G. (2021). Avian pathogenic *Escherichia coli* (APEC): An overview of virulence and pathogenesis factors, zoonotic potential, and control strategies. Pathogens.

[2] Kemmett K., Williams N., Chaloner G., Humphrey S., Wigley P., Humphrey T. (2014). The contribution of systemic *Escherichia coli* infection to the early mortalities of commercial broiler chickens. Avian Pathol..

[3] Sahin O., Zhang Q., Meitzler J.C., Harr B.S., Morishita T.Y., Mohan R. (2001). Prevalence, antigenic specificity, and bactericidal activity of poultry anti-*Campylobacter* maternal antibodies. Appl. Environ. Microbiol..

[4] Smith N.C., Wallach M., Miller C., Morgenstern R., Braun R., Eckert J. (1994). Maternal transmission of immunity to *Eimeria maxima*: Enzyme-linked immunosorbent assay analysis of protective antibodies induced by infection. Infect. Immun..

[5] Cardenas-Garcia S., Ferreri L., Wan Z., Carnaccini S., Geiger G., Obadan A.O., Hofacre C.L., Rajao D., Perez D.R. (2019). Maternally derived antibodies protect against challenge with highly pathogenic avian influenza virus of the H7N3 subtype. Vaccines.

[6] Wickramasuriya S.S., Park I., Lee K., Lee Y., Kim W.H., Nam H., Lillehoj H.S. (2022). Role of physiology, immunity, microbiota, and infectious diseases in the gut health of poultry. Vaccines.

[7] Keyburn A.L., Portela R.W., Ford M.E., Bannam T.L., Yan X.X., Rood J.I., Moore R.J. (2013). Maternal immunization with vaccines containing recombinant NetB toxin partially protects progeny chickens from necrotic enteritis. Vet. Res..

[8] Crouch C., Withanage G., De Haas V., Etore F., Francis M. (2010). Safety and efficacy of a maternal vaccine for the passive protection of broiler chicks against necrotic enteritis. Avian Pathol..

[9] Dórea F.C., Cole D.J., Hofacre C., Zamperini K., Mathis D., Doyle M.P., Lee M.D., Maurer J.J. (2010). Effect of *Salmonella* vaccination of breeder chickens on contamination of broiler chicken carcasses in integrated poultry operations. Appl. Environ. Microbiol..

[10] da Silva Teixeira M., Lages D.H., Alves V.V., da Silva Martins N.R., de Freitas Neto O.C. (2022). Assessment of maternal immunity against *Salmonella enterica* serovar Heidelberg in progeny of broiler breeders vaccinated with different formulations of bacterins. Avian Pathol..

[11] Wallach M., Smith N.C., Petracca M., Miller C.M., Eckert J., Braun R. (1995). *Eimeria maxima* gametocyte antigens: Potential use in a subunit maternal vaccine against coccidiosis in chickens. Vaccine.

[12] Wallach M.G., Ashash U., Michael A., Smith N.C. (2008). Field application of a subunit vaccine against an enteric protozoan disease. PLoS ONE.

[13] Raymond K.N., Dertz E.A., Kim S.S. (2003). Enterobactin: An archetype for microbial iron transport. Proc. Natl. Acad. Sci. USA.

[14] Caza M., Lépine F., Dozois C.M. (2011). Secretion, but not overall synthesis, of catecholate siderophores contributes to virulence of extraintestinal pathogenic *Escherichia coli*. Mol. Microbiol..

[15] Robinson A.E., Heffernan J.R., Henderson J.P. (2018). The iron hand of uropathogenic *Escherichia coli*: The role of transition metal control in virulence. Future Microbiol..

[16] Flo T.H., Smith K.D., Sato S., Rodriguez D.J., Holmes M.A., Strong R.K., Akira S., Aderem A. (2004). Lipocalin 2 mediates an innate immune response to bacterial infection by sequestrating iron. Nature.

[17] Xiao X., Yeoh B.S., Vijay-Kumar M. (2017). Lipocalin 2: An emerging player in iron homeostasis and inflammation. Annu. Rev. Nutr..

[18] Wang H., Zeng X., Mo Y., He B., Lin H., Lin J. (2019). Enterobactin-specific antibodies induced by a novel enterobactin conjugate vaccine. Appl. Environ. Microbiol..

[19] Zeng X., Wang H., Huang C., Logue C.M., Barbieri N.L., Nolan L.K., Lin J. (2021). Evaluation of the immunogenic response of a novel enterobactin conjugate vaccine in chickens for the production of enterobactin-specific egg yolk antibodies. Front. Immunol..

[20] Wang H., Zeng X., Lin J. (2020). Enterobactin-specific antibodies inhibit in vitro growth of different Gram-negative bacterial pathogens. Vaccine.

[21] Wang H., Cao L., Logue C.M., Barbieri N.L., Nolan L.K., Lin J. (2023). Evaluation of immunogenicity and efficacy of the enterobactin conjugate vaccine in protecting chickens from colibacillosis. Vaccine.

[22] Anderson M.T., Armstrong S.K. (2004). The BfeR regulator mediates enterobactin-inducible expression of *Bordetella* enterobactin utilization genes. J. Bacteriol..

[23] Zeng X., Lin J. (2017). Characterization of high affinity iron acquisition systems in *Campylobacter jejuni*. Campylobacter jejuni.

[24] Cui Y., Wang H., Guo F., Cao X., Wang X., Zeng X., Cui G., Lin J., Xu F. (2022). Monoclonal antibody-based indirect competitive ELISA for quantitative detection of Enterobacteriaceae siderophore enterobactin. Food Chem..

[25] Zhong Z., Yu Y., Jin S., Pan J. (2018). Effects of mixing eggs of different initial incubation time on the hatching pattern, chick embryonic development and post-hatch performance. PeerJ.

[26] Dziva F., Hauser H., Connor T.R., van Diemen P.M., Prescott G., Langridge G.C., Eckert S., Chaudhuri R.R., Ewers C., Mellata M. (2013). Sequencing and functional annotation of avian pathogenic *Escherichia coli* serogroup O78 strains reveal the evolution of *E. coli* lineages pathogenic for poultry via distinct mechanisms. Infect. Immun..

[27] Antao E.-M., Glodde S., Li G., Sharifi R., Homeier T., Laturnus C., Diehl I., Bethe A., Philipp H.-C., Preisinger R. (2008). The chicken as a natural model for extraintestinal infections caused by avian pathogenic *Escherichia coli* (APEC). Microb. Pathog..

[28] Nagy T.A., Moreland S.M., Andrews-Polymenis H., Detweiler C.S. (2013). The ferric enterobactin transporter Fep is required for persistent *Salmonella enterica* serovar typhimurium infection. Infect. Iimmun..

[29] Holden V.I., Breen P., Houle S., Dozois C.M., Bachman M.A. (2016). *Klebsiella pneumoniae* siderophores induce inflammation, bacterial dissemination, and HIF-1α stabilization during pneumonia. mBio.

[30] Fischbach M.A., Lin H., Liu D.R., Walsh C.T. (2006). How pathogenic bacteria evade mammalian sabotage in the battle for iron. Nat. Chem. Biol..

[31] Zeng X., Mo Y., Xu F., Lin J. (2013). Identification and characterization of a periplasmic trilactone esterase, Cee, revealed unique features of ferric enterobactin acquisition in *Campylobacter*. Mol. Microbiol..

[32] Schalk I.J., Perraud Q. (2022). *Pseudomonas aeruginosa* and its multiple strategies to access iron. Environ. Microbiol..

[33] Miethke M., Marahiel M.A. (2007). Siderophore-based iron acquisition and pathogen control. Microbiol. Mol. Biol. Rev..

[34] Lin J., Hogan J., Smith K. (1999). Growth responses of coliform bacteria to purified immunoglobulin G from cows immunized with ferric enterobactin receptor FepA. J. Dairy Sci..

[35] Cui Y., Guo F., Guo J., Cao X., Wang H., Yang B., Zhou H., Su X., Zeng X., Lin J. (2020). Immunization of chickens with the enterobactin conjugate vaccine reduced *Campylobacter jejuni* colonization in the intestine. Vaccines.

[36] Mellata M., Ameiss K., Mo H., Curtiss III R. (2010). Characterization of the contribution to virulence of three large plasmids of avian pathogenic *Escherichia coli* χ7122 (O78: K80: H9). Infect. Immun..

